# Brain connectivity alterations during sleep by closed-loop transcranial neurostimulation predict metamemory sensitivity

**DOI:** 10.1162/netn_a_00201

**Published:** 2021-08-30

**Authors:** Ryan J. Hubbard, Iman Zadeh, Aaron P. Jones, Bradley Robert, Natalie B. Bryant, Vincent P. Clark, Praveen K. Pilly

**Affiliations:** Center for Human-Machine Collaboration, Information and Systems Sciences Laboratory, HRL Laboratories, LLC, Malibu, CA, USA; Beckman Institute for Advanced Science and Technology, University of Illinois Urbana-Champaign, IL, USA; Center for Human-Machine Collaboration, Information and Systems Sciences Laboratory, HRL Laboratories, LLC, Malibu, CA, USA; Oracle Cloud Infrastructure, Oracle Corporation, 500 Oracle Parkway Redwood Shores, CA, USA; Psychology Clinical Neuroscience Center, Department of Psychology, The University of New Mexico, Albuquerque, NM, USA; Psychology Clinical Neuroscience Center, Department of Psychology, The University of New Mexico, Albuquerque, NM, USA; Psychology Clinical Neuroscience Center, Department of Psychology, The University of New Mexico, Albuquerque, NM, USA; Psychology Clinical Neuroscience Center, Department of Psychology, The University of New Mexico, Albuquerque, NM, USA; Center for Human-Machine Collaboration, Information and Systems Sciences Laboratory, HRL Laboratories, LLC, Malibu, CA, USA

**Keywords:** Brain stimulation, EEG, Metamemory, Functional connectivity, Graph theory, Sleep

## Abstract

Metamemory involves the ability to correctly judge the accuracy of our memories. The retrieval of memories can be improved using transcranial electrical stimulation (tES) during sleep, but evidence for improvements to metamemory sensitivity is limited. Applying tES can enhance sleep-dependent memory consolidation, which along with metamemory requires the coordination of activity across distributed neural systems, suggesting that examining functional connectivity is important for understanding these processes. Nevertheless, little research has examined how functional connectivity modulations relate to overnight changes in metamemory sensitivity. Here, we developed a closed-loop short-duration tES method, time-locked to up-states of ongoing slow-wave oscillations, to cue specific memory replays in humans. We measured electroencephalographic (EEG) coherence changes following stimulation pulses, and characterized network alterations with graph theoretic metrics. Using machine learning techniques, we show that pulsed tES elicited network changes in multiple frequency bands, including increased connectivity in the theta band and increased efficiency in the spindle band. Additionally, stimulation-induced changes in beta-band path length were predictive of overnight changes in metamemory sensitivity. These findings add new insights into the growing literature investigating increases in memory performance through brain stimulation during sleep, and highlight the importance of examining functional connectivity to explain its effects.

## Author Summary

Numerous studies have demonstrated a clear link between sleep and memory—namely, memories are consolidated during sleep, leading to more stable and long-lasting representations. We have previously shown that tagging episodes with specific patterns of brain stimulation during encoding and replaying those patterns during sleep can enhance this consolidation process to improve confidence and decision-making of memories (metamemory). Here, we extend this work to examine network-level brain changes that occur following stimulation during sleep that predict metamemory improvements. Using graph theoretic and machine-learning methods, we found that stimulation-induced changes in beta-band path length predicted overnight improvements in metamemory. This novel finding sheds new light on the neural mechanisms of memory consolidation and suggests potential applications for improving metamemory.

## INTRODUCTION

The brain has the remarkable ability to turn brief encounters and episodes, even “one-shot” encounters, into long lasting memories. This occurs through a process known as memory consolidation, in which memories in a labile state are replayed during sleep and converted to more stable representations. However, successful retrieval of memories involves control processes and decision-making, and even memories that are consolidated during sleep may be difficult to recall, or retrieved with little confidence in their veracity (Koriat & Goldsmith, 1996). Metamemory sensitivity, or an individual’s ability to judge the accuracy of their memories with confidence, plays a critical role in the usage of our memories. For instance, an eyewitness to a crime may have successfully encoded critical details of the episode, but may not be confident in their memory, leading to incorrect decisions (Luus & Wells, 1994; Memon et al., 2003; Sporer et al., 1995). Thus, improving not only memory retrieval, but also metamemory sensitivity, is of critical importance. Here, we investigated the improvement of sensitivity with an intervention while individuals slept.

During sleep, neuronal ensembles representing previously encoded memories are reactivated in both the hippocampus and neocortical areas (Euston et al., 2007; Ji & Wilson, 2007; Nádasdy et al., 1999; Sirota et al., 2003; Skaggs & McNaughton, 1996; Wilson & McNaughton, 1994). Memory replays are predominantly observed during slow-wave sleep, particularly during the positive phases, or up-states, of the ongoing 0.5–1.2 Hz oscillation (Lee & Wilson, 2002; Mölle & Born, 2011). Reactivations of encoding-specific neuronal patterns are accompanied by 12–15 Hz thalamo-cortical oscillatory activity known as spindles, as well as short-lived high-frequency bursts in the hippocampus called ripples (De Gennaro & Ferrara, 2003; Mölle et al., 2006). The intricate coordination of replays, spindles, and ripples is essential to facilitate the consolidation of memories into long-term storage, or to transfer memories from the hippocampus to the neocortex (McClelland et al., 1995; McGaugh, 2000; Rasch & Born, 2013; Staresina et al., 2015). Consolidation of memories not only may facilitate their later retrieval, but also may be related to the individual’s future confidence in them; namely, consolidation can strengthen memories and improve learning (Walker & Stickgold, 2004), and confidence in memories is related to memory fidelity (Dallenbach, 1913). Thus, targeting the consolidation process with an intervention could benefit not only retrieval success, but metamemory sensitivity.

Over the past decade, researchers have increasingly investigated ways of boosting memory consolidation processes through external manipulations. These intervention studies have shown that sleep-dependent memory consolidation can be enhanced in two ways. First, memory reactivations can be triggered during sleep by reexposing individuals to external sensory cues, such as odors or sounds that were present during encoding (Antony et al., 2012; Oudiette & Paller, 2013; Schreiner & Rasch, 2014; Rasch et al., 2007; Rudoy et al., 2009). This cued reactivation can lead to benefits in recall for the specific items previously associated with the cues. Second, multiple studies have shown that applying transcranial electrical stimulation (tES) at particular frequencies to the brain during sleep can potentiate endogenous electrophysiological processes, leading to facilitation of memory consolidation and subsequent recognition or recall (Ketz et al., 2018; Ladenbauer et al., 2016, 2017; Lustenberger et al., 2016; Marshall et al., 2006, 2004; Westerberg et al., 2015). These studies have demonstrated a general increase in memory retrieval performance following tES during sleep. Importantly, this stimulation-related benefit to memory could potentially be due to frequency-specific alterations in functional connectivity between brain regions (Krause et al., 2017).

To summarize, previous research indicates that retrieval of memories can be strengthened through neurostimulation during sleep, and would potentially suggest that individuals would also have greater metamemory sensitivity, or greater correlation in the accuracy and confidence for their memory decisions, for these episodes. Indeed, some research has demonstrated a relationship between healthy uninterrupted sleep and intact metamemory judgments (Daurat et al., 2010). However, this may not be the case, as other research has demonstrated dissociations between first-order decisions (recognition judgments) and second-order decisions (confidence judgments; Del Cul et al., 2009; Hebart et al., 2016; Rounis et al., 2010). This is important, as memory confidence can decrease over time (Shapira & Pansky, 2019), leading to errors in memory reporting and poorer decision-making. Neural stimulation of the prefrontal cortex has been shown to improve memory monitoring for general knowledge questions (Chua & Ahmed, 2016; Chua et al., 2017), and theta-burst stimulation to depress activity of the frontopolar cortex influenced metacognitive judgments (Ryals et al., 2016), suggesting tES techniques could be effective for improving and maintaining memory sensitivity for newly one-shot encoded episodes. Indeed, recent work in our lab has shown that unique spatiotemporal amplitude-modulated patterns (STAMPs) of tES can be used to boost the sleep consolidation and sensitivity of judgments of specific episodic memories acquired in immersive virtual reality (Pilly et al., 2020).

In this paper, we extend previous work on cueing memory reactivation by investigating changes in functional connectivity following short-duration tES patterns (namely, STAMPs) during sleep. Functional connectivity has previously been shown to be affected by tES during wake (Polanía et al., 2011, 2012), as well as by memory consolidation during sleep (Mölle et al., 2004). Additionally, consolidation of memories in the brain is thought to be a systems-level process, in that it is supported by a combination of short-range and long-range communication across brain structures (Staresina et al., 2015). This is likely similar for metamemory, as research has shown connectivity between a distributed network of brain areas, including the frontal cortex, precuneus, and hippocampus supports memory and metacognitive judgments (Baird et al., 2013; Molenberghs et al., 2016; Morales et al., 2018; Ren et al., 2018; Ye et al., 2019). Thus, understanding how changes in functional connectivity relate to this process is critical; however, to our knowledge no study to date has examined how changes in functional connectivity due to stimulation during sleep might affect or relate to memory consolidation and decision-making processes.

To examine changes in functional connectivity, we used measures of EEG coherence (Nunez, 1995), specifically the imaginary part of coherency (Nolte et al., 2004), and extracted features of connectivity from the coherence data with graph theoretical analyses (Bullmore & Sporns, 2009; Sporns, 2003). This approach models the functional connectivity of the brain as an interconnected graph, and allows for exploration of the relationship of network structure and function. While we were interested in connectivity changes in the Spindle band, we expanded the analysis to include several other frequency bands, as activity in many spectral bands have been related to memory processes (Hanslmayr & Staudigl, 2014; Hanslmayr et al., 2012; Lisman & Jensen, 2013). We then employed machine learning–based techniques to determine the important graph theoretic features for discriminating between Active and Sham stimulation conditions, as well as predicting overnight changes in episodic memory behavior. In this way, we provide novel insight into modulations in functional connectivity following pulsed tES that are related to changes in metamemory sensitivity for specific episodic memories.

## MATERIALS AND METHODS

The participants reported in this paper are the same group of participants from Pilly et al. (2020). They received unique brief spatiotemporal patterns of tES (namely, STAMPs) during encoding of episodic information, half of which were reapplied during up-states of slow-wave oscillations (SWOs) during subsequent nights to cue reactivation of the specific associated memories (Active condition). At another point in time, the same individuals also performed the memory task without brain stimulation (Sham condition). Therefore, we were interested not only in the changes in functional connectivity that differed between the Active and Sham stimulation conditions, but also in the connectivity changes following STAMPs that were related to changes in the recall of specific episodic memories from pre- to postsleep.

### Participants

A total of 30 healthy participants completed the experiment, who were recruited using flyers placed around campus of the University of New Mexico and surrounding community, and received monetary compensation upon completion of the study. Of these, six participants were excluded from the analyses due to either equipment failure to stimulate during an Active night, or noncompliance in following task instructions. The sleep EEG data from additional six participants could not be used to calculate functional connectivity measures due to excessive artifacts, leading to the inclusion of *N* = 18 participants in the final analysis and reporting. All participants provided signed informed consent to participate in the study, which was approved by the Chesapeake Institutional Review Board. All participants were native English speakers, had normal or corrected-to-normal hearing and vision, and had no history of neurological or psychiatric disorder, or drug abuse.

### Behavioral Paradigm and Procedure

**Figure 1.**
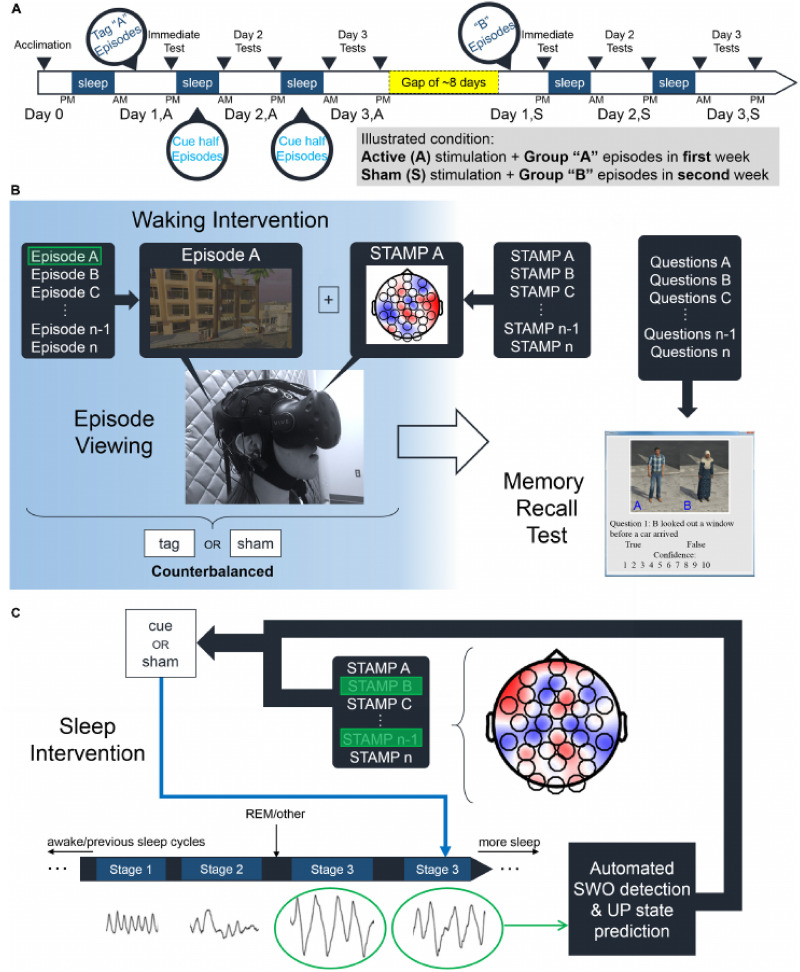
Outline of experimental procedure, reprinted with permission from Pilly et al. (2020). (A) After an acclimation night, participants viewed episodes accompanied by either Active or Sham spatiotemporal amplitude-modulated patterns (STAMPs) stimulation. After a gap of time, participants viewed more episodes accompanied by the other stimulation condition. (B) Encoding of episodes occurred in a virtual environment. STAMP stimulation occurred during episode viewing. Following encoding, memory recall tests were given over a period of 2 days on details of the episodes. (C) During sleep, STAMPs were delivered during up-states of slow-wave oscillations (SWOs) to cue reactivation of specific episodes. Scalp topography shows the amplitudes of current for an illustrative STAMP (red signifies positive and blue signifies negative currents).

An outline of the experimental procedure is presented in Figure 1. The experiment was made up of an acclimation period to train participants and let them sleep in the lab, followed by two experimental nights involving learning and testing. The acclimation night was only to allow participants to become accustomed to sleeping in the lab, and EEG data was not recorded or analyzed from this period. Participants encoded information on the first experimental night. The memory task consisted of viewing virtual reality episodes administered with an HTC Vive VR headset, followed by several memory recall tests on details from the episodes. Participants encoded 14 virtual reality vignettes, each lasting about a minute, depicting a series of events with two or more characters committing some action around an apartment complex. An additional vignette that was longer in length was used to train participants during the acclimation period. Participants were tested on their memory for the vignettes across five test sessions administered over a period of 48 hr with a non-VR computerized task built in MATLAB. For each vignette, 10 test items were constructed that consisted of True/False statements on specific aspects of the vignette. Each of the five test lists contained 28 items, 2 for each vignette. Participants reported whether the test statements were True/False, as well as the confidence of their recall on a scale of 1–10. Participants slept in the lab for each of the three nights.

The procedure consisted of four experimental sessions and one acclimation session. In the acclimation session, participants viewed a long practice vignette and answered practice test questions, and afterwards slept in the lab. In the following first experimental session (Session 1), participants encoded the experimental stimuli. Half of the participants received unique tES with unique STAMPs during viewing of vignettes (Active condition), whereas the other half did not receive any stimulation (Sham condition). The participants received the opposite stimulation condition over two additional experimental nights separated by about 1 week. After completing the vignette viewing procedure, participants were given their first memory recall test, and then slept overnight in the lab. This night, and not the acclimation night, was considered “Night 1.” The participants in the Active condition received half of the STAMPs during the night to cue consolidation of specific memories during the night. In contrast, participants in the Sham condition did not receive stimulation during the night. Note that this study design allowed us to compare memory performance for episodes that received STAMP stimulation during encoding and sleep (Tag & Cue) to episodes that only received stimulation at encoding (Tag & No Cue). For the connectivity analyses, we focused on the Tag & Cue and Sham (no encoding or sleep stimulation) conditions. After the participant awoke, they were given a second memory test, and the experimental session concluded.

The second experimental session (Session 2) occurred in the evening after the first session. Participants were given a third memory test in the evening, went to sleep, and received another memory test after waking. This second period of sleeping in the lab was considered “Night 2.” Active group participants once again received STAMPs during the night. The final memory test was administered later in the evening. For the opposite stimulation condition (Sessions 3 and 4), the participants viewed a new set of 14 vignettes and were administered corresponding memory recall tests over 2 days. In this way, the experiment was a within-subjects manipulation, with the Active and Sham conditions occurring approximately 1 week apart. The assignment of the stimulation condition order (Active first vs. Sham first), as well as the assignment of vignettes, were counterbalanced across participants. The analyses reported in this paper focus on Night 2, where the largest behavioral effects of STAMP stimulation were found (see Pilly et al., 2020, for further details).

### Electrophysiological Recordings and Transcranial Stimulation

EEG data was collected and stimulation was applied simultaneously with a prototype StarStim64 from Neuroelectrics, Inc., with a 64-channel neoprene head cap including 32 EEG channels and 32 stimulation channels. EEG channels were placed in a montage according to the 10-10 system. EEG data was collected from 23 of the 32 sites sampled at 500 Hz and referenced to channel Fz. The additional 9 electrodes recorded electrooculogram, electromyogram, and electrocardiogram to allow for detection of sleep stages. No online hardware filtering, except line noise (60 Hz) filtering, was applied during collection.

Active transcranial stimulation was delivered in the form of unique arrays of spatiotemporal currents across the 32 stimulation electrodes. The total positive electrical current was set to be under 2.5 mA, with each individual channel’s current under 1.5 mA and over 150 uA. The patterns of stimulation were created through gradient descent optimization to maximize orthogonality across the induced electric fields in the 3-D volume of an adult human head template by penalizing correlations and anticorrelations. During the experiment, for the Active condition, each of the 14 vignettes were randomly associated or “tagged” with a different pattern of stimulation, and custom templates for each pattern were programmed in the Neuroelectrics stimulation control software, CoreGUI. Only half of these tES patterns were reapplied during the night to cue memory reactivation (Tag & Cue), and behavioral analyses focused on memory recall test responses for the targeted vignettes. The unique patterns were primarily designed to cue specific memories, not to alter neural activity in specific ways; thus, brain responses and connectivity changes to individual STAMPs were not analyzed, and instead were combined to increase trial counts.

### Slow-Wave Oscillation Detection

Stimulation was automatically delivered time-locked to up-states of SWOs with a closed-loop detection algorithm, adapted from a previously developed algorithm (Cox et al., 2014). During sleep, incoming EEG data from 13 fronto-parieto-central channels (Cz, FC1, FC2, CP1, CP2, Fz, C4, Pz, C3, F3, F4, P3, and P4) were stored in a running 5-s buffer that was continuously updated. To clean the data, moving average subtraction with a 1-s window was applied, and any channels exceeding 500 μV min-to-max amplitude across the buffer period were rejected. The remaining channels were averaged together to create a virtual channel for robust SWO detection. Power within the slow-wave band (0.5–1.2 Hz) was compared to total broadband power (0.1–250 Hz), and if this ratio was greater than 0.3, then the precise peak of power in the slow-wave band (the center frequency) was computed. The virtual channel was then filtered with a second-order Butterworth bandpass filter with a 1-Hz bandwidth around the center frequency (with a minimum lower cutoff of 0.1 Hz), and a Hilbert transform was applied. The phase was shifted back 90 degrees, and the imaginary component was extracted, yielding the instantaneous phase. A sine wave at the center frequency with optimized phase, offset, and amplitude of the filtered signal was then generated. The sine wave was projected forward in time to determine the next point of zero phase (i.e., the start of the up-state), and stimulation was started at this time point and lasted for the predicted up-state with ramp up and ramp down times of 100 ms. During Sham nights, stimulation time points were recorded, but no stimulation was delivered. Thus, the comparison of Active and Sham nights were at the same time points of the SWO, with the only difference being the delivery of STAMP stimulation.

Validation of up-state detection was performed on data from Sham nights to avoid artifacts produced by STAMPs in Active nights. Markers were extracted from the time points of up-states predicted by the sine wave fit. In some instances, due to hardware delay, timed stimulation was moved to the up-state following the immediately detected up-state, which may have increased the variability of phases to some degree. Epochs time-locked to stimulation onset markers (−5 to +5 s) were extracted from the raw EEG data and bandpass filtered in the 0.5–1.2 Hz range. Phase values at each onset marker time point were calculated using the Hilbert transform. Mean phase values across trials were calculated for each participant, and these mean phases were submitted to a v-test testing for a difference from 0 degrees (Berens, 2009). Figures and statistics on the validation of the closed-loop algorithm are presented in the Supporting Information.

### EEG Data Processing and Graph Theoretic Analysis

EEG signals were analyzed off-line with EEGLAB and custom MATLAB scripts. To clean the data and separate sources of noise from brain signal, the EEG recordings were down-sampled to 250 Hz, rereferenced to the average of all electrodes, and bandpass filtered using a Butterworth filter (0.1–70 Hz, 24 dB/oct). Ocular and nonneural artifacts were identified and manually removed using the independent component analysis (ICA) method in EEGLAB and its built-in infomax algorithm (Delorme & Makeig, 2004). The artifact-free data were segmented in epochs containing −6.4 to −2.4 s prior to the onset of stimulation as a baseline window, and 3 to 7 s following the offset of stimulation as an analysis window. For each epoch, the power spectrum at each channel was calculated by applying the Welch spectral estimation method on 2048-ms segments with a 1,024-ms (50%) overlap, with each segment tapered by a Hamming window.

Functional connectivity and graph theoretic analyses were performed with the BioNeCt Toolbox, a custom MATLAB toolbox for brain connectivity analysis (Casanova, El-Baz, & Suri, 2017). Functional brain connectivity between brain regions were estimated by computing the cross-correlation in the frequency domain between EEG signals (Bowyer, 2016; Mohammad-Rezazadeh et al., 2016; Nolte et al., 2004). Among the various metrics of functional connectivity, imaginary coherence (iCoh) exclusively detects “true” interactions between EEG signals occurring within a certain time delay, thus ignoring instantaneous interaction between neighboring electrodes likely produced by volume conduction of electrical activity from a common brain source (Bullmore & Sporns, 2009; Fallani et al., 2014; Mohammad-Rezazadeh et al., 2016). iCoh values were computed for all possible electrode pairs in the following frequency bands: delta (1–4 Hz), theta (4–8 Hz), alpha (8–12 Hz), spindle (12–15 Hz), beta (16–30 Hz), and low gamma (40–50 Hz). These analyses were performed on both the Active and Sham data. Additionally, iCoh values were computed for both the poststimulation and prestimulation time windows, and the prestimulation window values were subtracted from the poststimulation windows values for baseline correction.

The high multidimensionality of the iCoh measures was disentangled with a graph theoretic approach. Graph theoretic metrics provide information on the degree of network segregation (i.e., the tendency of brain regions to form local clusters with dense functional interconnections) and network integration and efficiency (i.e., the capacity of the network to become interconnected and efficiently exchange information between brain regions; Bullmore & Sporns, 2009; Fallani et al., 2014; Mohammad-Rezazadeh et al., 2016). The following commonly used graph measures were calculated for all of the above mentioned frequency bands in the pre- and poststimulation periods: average clustering coefficient (the probability of neighboring nodes being connected to each other, reflecting local connectedness); global efficiency (how efficient the network is in transferring information); characteristic path length, radius, and diameter (the average number of edges along the shortest paths, the minimum possible distance, and the largest possible distance, between all possible pairs of nodes, respectively); modularity (the degree to which the brain network is segregated into subnetworks or modules); assortativity (the proportion of nodes that are attached to other nodes with similar degrees vs. dissimilar degrees); density (the number of edges divided by the number of nodes in the graph); and mean coherence (the average coherence between all pairs of nodes).

### Classification and Regression Analyses

The resulting data consisted of 48 functional connectivity features (8 graph theoretic metrics × 6 frequency bands) for both the Active and Sham stimulation conditions for each participant. More specifically, connectivity features were extracted following stimulation on Active nights in which STAMP stimulation occurred, as well as during Sham nights in which no stimulation occurred. To investigate what connectivity changes are predictive of stimulation condition, as well as relate to behavior, we implemented a participant-level classification and regression cross-validation analysis, in which data from a subset of participants were left out as test data to predict. To reduce variance, we ran 35 folds in which one to three participants were left out at a time. This repeated random split method of sampling is preferred over the commonly implemented “leave one out” method, which can lead to unstable and biased estimates of variance (Scheinost et al., 2019; Varoquaux et al., 2017). We chose to take this data-driven approach not only due to the large feature space of the data, but also due to the novelty of the investigation. Few studies to date have examined relationships between graph theoretic metrics of brain connectivity and metamemory sensitivity, and none have examined how this changes with brain stimulation. Thus, we would potentially miss important relationships between functional connectivity and metamemory by only examining certain frequency bands (e.g., theta) or measures (e.g., mean coherence) based on previous findings in the literature.

**Figure 2.**
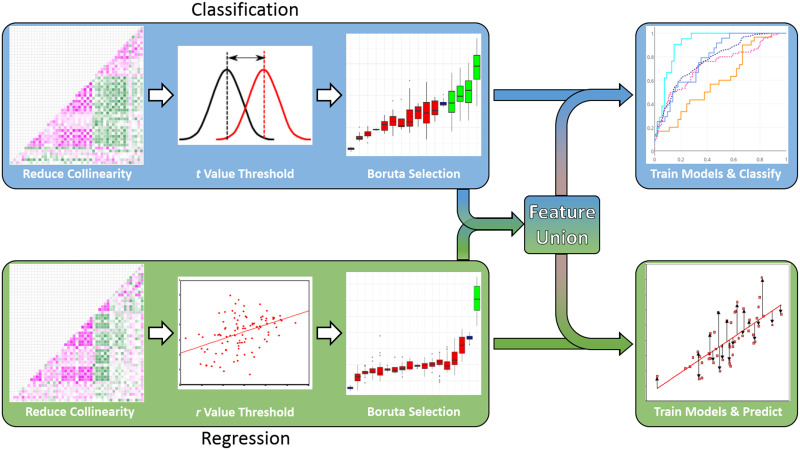
Outline of analysis pipeline. Feature selection is performed for both the classification of Active vs. Sham condition, as well as the prediction of overnight behavioral changes in the Active condition. The top features from each selection process are also combined and tested for the classification and regression analyses.

A diagram outlining the analysis pipeline is presented in Figure 2. For each fold, a feature selection method was run on the training data to find graph theoretic features (poststimulation baseline corrected with prestimulation) that discriminated between Active and Sham stimulation conditions. Many features were highly correlated with one another, which can be seen in Supporting Information Figure S2. Thus, first highly collinear variables were removed by finding pairs of features with high correlations (threshold *r* value of 0.8) and removing the variable with the higher mean absolute correlation with the other variables. Additional information on removed variables due to high collinearity can be found in the Supporting Information. Then, a dependent *t* test (as the study was a within-subjects design) was run on each feature, testing the difference between Active and Sham conditions. Variables with low absolute *t* values (threshold of 1) were removed, with the rationale that the difference between these variables was minimal.

The remaining features were entered into the Boruta feature selection algorithm (Kursa & Rudnicki, 2010). Boruta is a permutation-based selection method, in which each feature, as well as a copy of the feature with its values shuffled across participants (called a “shadow feature”), is entered into a random forest classifier, and the importance of each feature is determined as a *Z* score. The importance of the true features must be higher than the max *Z* score of the shadow features to be considered significantly important. This procedure is repeated several times with different permutations of the shadow features to obtain a distribution of values to which the importance values of the actual features can be compared. Thus, Boruta is an all-relevant feature selection method—essentially, all or none of the variables could be selected by the process. Only features that had higher *Z* scores than the shadow features were selected. This algorithm has been shown to improve performance in finding meaningful features compared to other feature selection algorithms (Degenhardt et al., 2017; Kursa, 2017).

The features selected by Boruta were then entered into a logistic regression model predicting Active versus Sham condition. We also ran a regularized logistic regression with L_2_ regularization (ridge regression), in which the optimal λ parameter was determined through cross-validation within the training data. Lastly, we also ran a logistic regression classification using only the most important variable selected by the Boruta algorithm. After fitting the model parameters on the training data, the models were used to predict the left-out test data, yielding estimates of probability for each test example either belonging to the Active or Sham condition. These probabilities were concatenated across folds, and used to calculate relative operating characteristic curves, from which we derived area under the curve (AUC) values (Fawcett, 2006). AUC is a more sensitive measure of classifier performance than simply raw accuracy, and thus is reported here.

Within the same cross-validation fold, the training data was used to predict changes in memory sensitivity for the targeted vignettes (namely, Tag & Cue) from the Night 2 presleep test to the postsleep test in the Active condition. The measure of behavioral performance was AUC of the type-2 relative operating characteristic curve, a bias-free nonparametric measure of metacognitive sensitivity, with greater AUC reflecting greater memory sensitivity (Fleming & Lau, 2014; Galvin et al., 2003). Here, the models predicted the overnight change in AUC in order to tie changes in connectivity with changes in memory sensitivity. All of the connectivity features were entered into a similar feature selection process as the classification analysis. Highly collinear features were removed as before. Instead of a *t* test, the correlation between each feature and the overnight AUC change was computed, and features with low absolute *r* values (threshold of 0.1) were removed. The remaining features were run through the Boruta algorithm, this time predicting overnight AUC changes. These features, as well as just the top feature, were entered into linear regression and L_2_ regularized regression models predicting left-out test data. Root-mean-square error (RMSE) values were calculated, and average RMSE values across folds were compared to RMSE values from predictions from a linear regression with only the intercept.

As a final analysis, we took Boruta-selected features from the classification analysis, as well as the Boruta-selected features from the regression analysis, and reran classification and regression with the combined feature set, as well as the top feature from each set. AUC values from classification analyses and RMSE values from regression analyses are reported for each feature set.

## RESULTS

### Behavioral Effects

As reported in Pilly et al. (2020), STAMP stimulation did not lead to a significant improvement of overall memory recall accuracy. However, STAMP stimulation led to improved metamemory sensitivity on Day 3 of the experiment (following Night 2). Specifically, dependent *t* tests comparing metamemory sensitivity across conditions revealed that sensitivity in the Tag & Cue condition was significantly greater than both the Tag & No Cue condition [*t*(23) = 3.51, adjusted *p* < 0.01, Holm–Bonferroni correction for two comparisons; paired-sample Cohen’s *d* = 0.72] and Sham condition [*t*(23) = 2.089, adjusted *p* = 0.048, Holm–Bonferroni correction for two comparisons; paired-sample Cohen’s *d* = 0.43]. A significant improvement in metamemory sensitivity was only found on Day 3 of the experiment. Thus, the application of STAMPs during SWOs in the two nights following one-shot viewing led to specific enhancement of metamemory for the episodes that were both tagged and cued.

### Global Coherence Changes

As a first pass, we examined overall changes in mean coherence across the scalp due to STAMP stimulation to determine if stimulation led to any measurable changes in connectivity. Additionally, we sought to replicate previous work and assess whether stimulation would produce similar changes in coherence as learning during sleep (Mölle et al., 2004). Previous work (Mölle et al., 2004) reported modulation of delta, spindle, and gamma band coherence during sleep following learning; in contrast, other work (Polanía et al., 2011) demonstrated increased connectivity across all frequency bands following tDCS stimulation. Therefore, we hypothesized that STAMP stimulation would increase mean coherence compared to the Sham condition in in the delta, spindle, and gamma bands, but examined differences in other bands as well with conservative statistical testing. Mean iCoh values in all frequency bands were computed for Active and Sham baseline-corrected data in order to investigate global connectivity changes, and thus one-tailed *t* tests were conducted on mean coherence values in each frequency band, and were corrected for using a false discovery rate of *p* < 0.05 (Benjamini & Hochberg, 1995). Coherence maps showing greater connectivity in the Active condition across the scalp were visualized by setting the visualization threshold to the maximum of the Sham condition for each frequency band, essentially subtracting the Sham condition.

**Figure 3.**
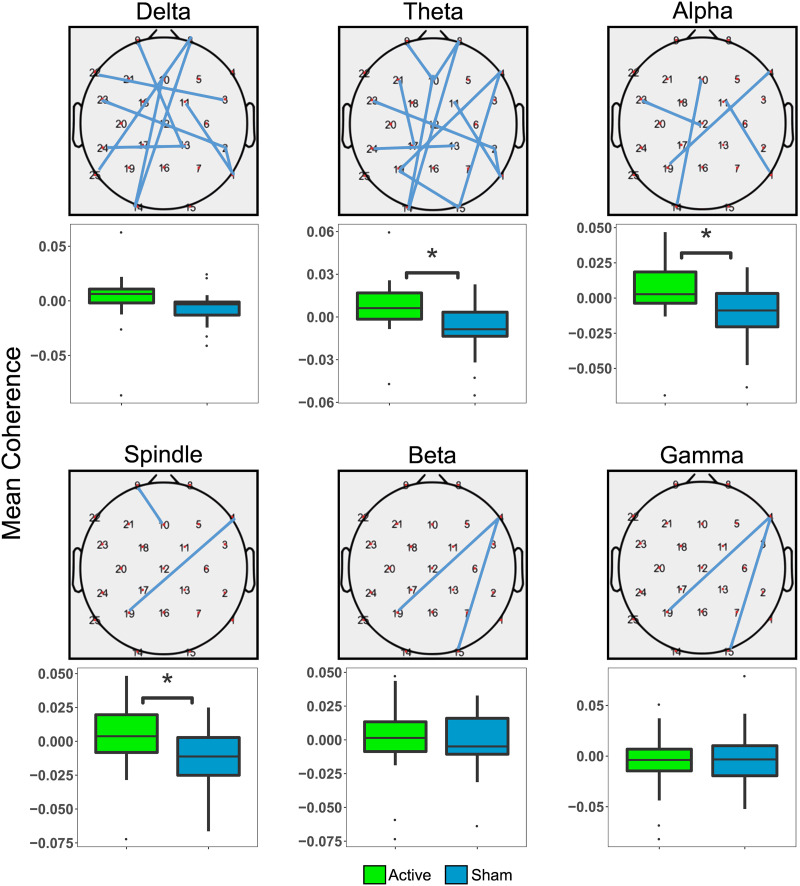
Overall changes in mean coherence across the scalp induced by STAMPs. For each frequency band, the scalp plot shows the active mean coherence data thresholded by the maximum coherence of the Sham data. The boxplot shows the mean coherence change from the baseline period to the poststimulation period across the entire scalp across participants for both the Active and Sham conditions, and the * symbol designates a significant difference (*p* < 0.05).

Stimulation-induced mean coherence changes in different frequency bands are plotted in Figure 3. The topography plots display the coherence between pairs of electrodes in the Active condition, thresholded by the coherence in the Sham condition, whereas the boxplots show nonthresholded mean coherence values across the entire scalp. The analysis of overall changes in coherence across the scalp revealed that STAMPs produced significant increases in mean coherence in multiple frequency bands. EEG coherence was significantly increased following STAMPs in the theta, alpha, and spindle bands (*p* < 0.05) compared to the Sham condition. Mean coherences in the delta, beta, and gamma bands did not significantly differ between the stimulation conditions. Thus, STAMP stimulation induced changes in brain connectivity in specific frequency bands.

### Classification and Behavioral Prediction

**Figure 4.**
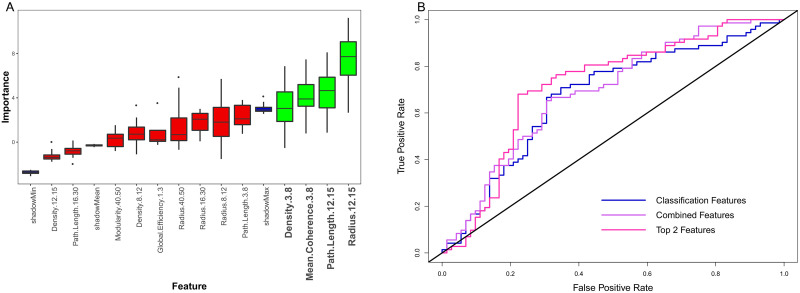
Classification results. (A) Boruta feature selection results, with red boxes showing rejected features, green boxes showing selected features, and blue boxes showing shadow feature statistics. The selected features are in the theta and spindle bands. (B) Relative operating characteristic curves for Active vs. Sham classification with different feature sets. All feature sets performed above chance accuracy, but the top classification and regression features performed the best.

Results from the classification analysis are plotted in Figure 4 and outlined in Table 1. Average importance values across folds for Boruta-selected features are plotted on the left, with features consistently considered important highlighted in green (significantly above the average shadow max). Across folds, radius in the spindle band (12–15 Hz) was consistently selected as the most important feature. Other important features included: path length (12–15 Hz) and mean coherence and density (3–8 Hz). ROC curves of classifier performance are plotted on the right. As outlined in Mason and Graham (2002), Mann–Whitney *U* tests were conducted on each classifier to determine if classifier performance was statistically significant above chance (AUC = 0.5). All tests reported significant *p* values (*p* < 0.001). We additionally calculated 95% confidence intervals using a stratified bootstrap resampling approach with 10,000 bootstrap replications, which further demonstrated above chance classification performance (Carpenter & Bithell, 2000). Thus, classification accuracy was above chance, and taking the top variables from classification and regression analyses (path length in 16–30 Hz and radius in 12–15 Hz) led to the best classification performance.

**Table 1.** AUC values and 95% bootstrap confidence intervals for classification using different feature sets

**Feature Set**	**AUC**
Selected classification features	0.671 (0.581–0.756)
Combined classification and regression features	0.689 (0.601–0.774)
Top classification and regression features	**0.715 (0.625–0.800)**

As a follow-up analysis, the four features selected by the Boruta algorithm for classification were submitted to *t* tests, testing the difference in connectivity between the Active and Sham conditions. The resultant *p* values were corrected with a false discovery rate set at 0.05. The corrected *p* values for 12–15 Hz path length and radius, and 3–8 Hz mean coherence were significant (*p* = 0.029); 3–8 Hz density was also significant at alpha level of significance (*p* = 0.05). Thus, connectivity features that were significantly altered following Active stimulation compared to Sham stimulation were found by the feature selection pipeline. Path length and radius features from pre- to poststimulation were significantly decreased in the Active condition compared to Sham, whereas mean coherence and density features were significantly increased compared to Sham. Boxplots showing these features for both conditions are presented in Supporting Information Figure S3.

**Figure 5.**
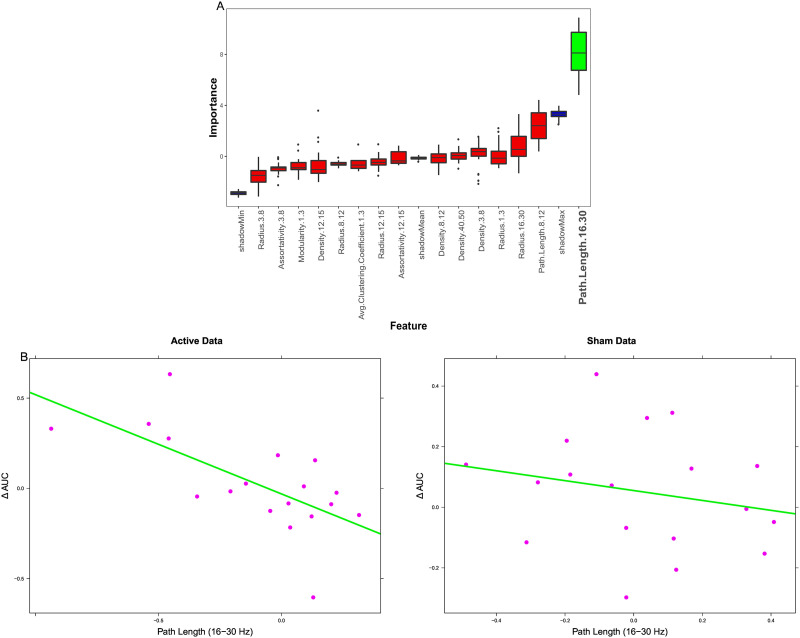
Regression results. (A) Boruta feature selection results, with red boxes showing rejected features, green boxes showing selected features, and blue boxes showing shadow feature statistics. Beta path length is consistently selected across folds. (B) Relationship between beta path length (*x*-axis) and overnight AUC change (*y*-axis) in the Active (left) and Sham (right) conditions. The relationship is significant in the Active condition, not in Sham condition.

Results from the behavioral prediction analysis are plotted in Figure 5 and outlined in Table 2. Here, changes in neural measures following stimulation were used as features to predict change in recall test metamemory sensitivity (AUC) for both the Tag & Cue and Sham conditions from Day 2 to Day 3 in order to identify brain-behavior relationships. The importance values from Boruta for graph theoretic features are plotted on the left—clearly, path length in the beta band (16–30 Hz) was considered highly important for predicting changes in metamemory sensitivity, and the only feature selected. The relationship between stimulation-induced change in 16–30 Hz path length and overnight change in AUC performance for both the Active (Tag & Cue) data and Sham conditions are plotted on the right. The correlation between change in beta-band path length and change in AUC was significant for the Active condition (*r* = −0.66, *p* = 0.003), indicating that individuals with decreased path length following STAMPs tended to show a positive change in memory performance. This relationship was not significant for the Sham condition (*r* = −0.22, *p* = 0.39).

**Table 2.** RMSE values for regression using different feature sets

**Feature Set**	**RMSE**
Base model (intercept only)	0.249
Selected regression features	0.226
Top regression feature	**0.208**
Combined classification and regression features	0.240
Top classification and regression features	0.215

Based on the outcome of these analyses, follow-up linear regressions were ran predicting overnight change in metacognitive sensitivity using path length in the 16–30 Hz band for both the Active data and the Sham data. For the Active data, 16–30 Hz path length significantly predicted AUC change (*t* = −3.56, *p* = 0.003, adj. *R*^2^ = 0.41). For the Sham data, 16–30 Hz path length was not a significant predictor (*t* = −0.88, *p* = 0.39, adj. *R*^2^ = −0.01). Additional analyses to compare the Active and Sham conditions were also performed and are outlined further in the Supporting Information.

## DISCUSSION

In the present experiment, participants engaged in an episodic memory task while receiving unique spatiotemporal patterns of transcranial electrical stimulation, and a subset of the patterns were reapplied during up-states of SWOs in subsequent nights to cue reactivation of specific associated memories (see Pilly et al., 2020). Here, we report changes in functional brain connectivity induced by these tES patterns during sleep that were predictive of changes in metamemory sensitivity from pre- to postsleep. Using machine learning techniques to identify important features for classifying between Active and Sham stimulation, we found that connectivity features in the theta and spindle bands significantly differed between the two conditions. Additionally, we found that changes in path length in the beta band predicted memory sensitivity changes, in that performance increases across the night were related to beta path length decreases following stimulation. Taken together, these results contribute to a growing body of research investigating the neural processes underlying memory reactivations and consolidation during sleep, and suggest that modulation of path length at specific frequencies during the consolidation process may lead to higher confidence and better decision-making for newly consolidated memories.

The short bursts of tES, or STAMPs, utilized in the current experiment increased overall scalp coherence in lower frequency bands—namely, in the theta, alpha, and spindle bands, or essentially from 3–15 Hz. Interestingly, changes in spindle-band connectivity during sleep was also found following learning; however, changes in connectivity were not found in the delta or gamma bands following STAMP stimulation, which is somewhat at odds with previous experimental work (Mölle et al., 2004). However, the stimulation protocol used here largely differed from previous studies—namely, we used short bursts of spatiotemporally distributed electrical currents that were primarily designed to cue specific memories due to their uniqueness and association with the encoding period, not modulate or entrain specific oscillations. Importantly, activity in the reported frequency bands, particularly the theta and spindle bands, has been shown to be related to the memory consolidation process (Staresina et al., 2015). Thus, STAMPs may have modulated the ongoing processes of memory reactivation or consolidation during sleep, leading to the changes in connectivity in the observed frequency bands.

Recent work has questioned the effectiveness of tES in significantly affecting behavior (Horvath et al., 2015), as well as in its ability to modulate neural firing patterns or oscillatory activity (Lafon et al., 2017; Vöröslakos et al., 2018). However, other studies have demonstrated measurable neural changes in humans and nonhuman primates following tES, even at lower intensity levels (Huang et al., 2017; Krause et al., 2017; Opitz et al., 2016). A particular concern is the ability of tES, specifically alternating current stimulation, to entrain neural oscillations to a specific frequency. Here, we do not claim that the reported electrophysiological changes are due to oscillatory entrainment to the short bursts of stimulation, or that we are inducing these changes de novo, but rather that the STAMPs act to boost ongoing memory consolidation processes, which occurs through subtle modulations of network-level connectivity. We would argue that the observed connectivity patterns during sleep are natural phenomena that occur and lead to memory consolidation, and STAMP stimulation acted upon these preexisting processes to boost metamemory sensitivity.

The reported modulation of graph theoretic metrics in the theta and spindle bands following STAMPs is in line with previous research implicating these oscillations in the processes of memory reactivation and consolidation. Targeted memory reactivation during sleep with auditory cues elicits increases in theta and spindle power (Ong et al., 2016; Schreiner et al., 2015; Schreiner & Rasch, 2014). Numerous studies have also shown the importance of theta-band activity for memory encoding and retrieval during wake (Backus et al., 2016; Lega et al., 2012; Nyhus & Curran, 2010); in particular, theta oscillations in the hippocampus are critical for episodic memory encoding (Hasselmo et al., 2002; Lin et al., 2017). Similarly, thalamocortical spindles are related to episodic memory (Van Der Helm et al., 2011) and play an important role in the process of memory consolidation, potentially facilitating the transfer of newly encoded information in the medial temporal lobes to stable representations in the cerebral cortex (Sirota et al., 2003; Staresina et al., 2015). During sleep, Theta activity may be indicative of the memory reactivation process, whereas spindle activity may reflect the active consolidation of the reactivated memories. Here, STAMPs may have cued reactivation of the associated memories, leading to boosted consolidation of this information. This consolidation led to improved confidence for memories downstream.

The theta-band graph theoretic measures that were significantly modulated by STAMPs were mean coherence and density. Mean coherence refers to the average coherence across all vertices, while density is calculated as the number of connections in the graph, normalized by the total possible number of connections (Sporns, 2003). Changes in these measures suggest that STAMPs increased overall coherence in the theta band between channels, leading to a greater number of edges above the cutoff threshold and thus a higher average connection density. This greater coherence and density may reflect an increase in the exchange of information in the theta band across neural areas following STAMPs. This is in line with previous findings implicating increases in theta coherence between the hippocampus and rhinal cortex (Fell et al., 2003), as well as frontal cortex (Anderson et al., 2009; Benchenane et al., 2010), as important for learning and memory, and potentially critical for memory reactivations (Carr et al., 2011).

The spindle-band graph theoretic measures that significantly differed following STAMP stimulation were path length and radius. These measures are related—radius refers to the minimum eccentricity of the graph and path length refers to the graph’s average eccentricity, where the eccentricity of a node is defined as the maximum distance to any other node (Sporns, 2003). Path length is often considered a measure of network integration or efficiency of information transfer, with smaller path lengths reflecting greater efficiency (Bullmore & Sporns, 2009; Reijneveld et al., 2007). Here, STAMPs led to an average decrease in path length and radius in the spindle band, meaning the average and minimum eccentricity were decreased, and efficiency of information transfer was increased. This may reflect a reorganization of the network toward a small-world network (Watts & Strogatz, 1998), a near optimal structure between a perfectly ordered and randomly organized system, allowing for greater flexibility and synchronization of neural activity (Barahona & Pecora, 2002; Bassett & Bullmore, 2006; Masuda & Aihara, 2004). Changes in “small-worldness” are often measured as changes in path length without concomitant changes in local clustering, or cluster coefficient. Thus, the observed change in path length may suggest boosted synchronization between brain areas, leading to more efficient information transfer by spindles. Note that effects in the theta band were more closely related to overall changes in mean coherence—essentially, changes in the magnitude of connectivity as opposed to the efficiency. While the data here is unable to establish a causal link between these changes, a hypothetical relationship is that STAMPs caused greater connectivity in the theta band (Fell et al., 2003), and Spindle information transfer efficiency, measured as path length, was ramped up to compensate for this increase, leading to increased memory reactivation.

Across studies, there is high variability in the frequency bands chosen to delineate slow and fast spindles (Ayoub et al., 2013; Holz et al., 2012; Schabus et al., 2007), and recent evidence suggests these delineations may in fact vary in each individual (Cox et al., 2017). We selected 8–12 Hz and 12–15 Hz as bands to separately investigate slow versus fast spindles, and our results suggest that connectivity in the fast spindle band was specifically modulated by STAMPs. However, there may be variability in spindle-band delineations across the participants in the experiment, leading to overlap of slow and fast spindle activity in these bands. In our data, path lengths in the 8–12 Hz and 12–15 Hz bands were highly correlated (*r* = 0.88), suggesting a high degree of overlap between these frequency bands. Thus, we claim here that spindle activity was modulated by STAMPs, but further analysis will be required to specifically delineate between fast and slow spindles. Additionally, we used a frequency band–based approach to accommodate the connectivity and machine learning analysis we implemented; however, spindles are transient events that occur briefly in time. While the approach here likely captured activity related to spindles, a more focused event-based approach in which specific spindle events are identified may further elucidate their role in metacognition.

The results of the regression analysis showed that changes in path length in the beta band were related to changes in memory sensitivity from presleep to postsleep in the Active condition. The reported relationship was in a sensible direction, with lower path length (greater efficiency) related to improvements in metacognitive performance. Note that beta path length did not, however, significantly differ between the Active and Sham conditions, suggesting the effect captures more fine-grained individual differences, in which individuals that exhibit network reorganization toward greater efficiency following STAMP stimulation show greater overnight metamemory improvement than those that do not. This is discussed further in the Supporting Information. This finding is fairly novel, as no prior study to our knowledge has examined changes in functional connectivity following stimulation during sleep that relate to behavioral changes. Studies investigating visuomotor learning and performance, such as with mirror tracing tasks, have reported changes in beta-band synchronization and connectivity during and following the task (Classen et al., 1998; Kilner et al., 2004; Piantoni et al., 2015); however, studies examining graph theoretic connectivity relationships to episodic memory performance, which was tested in the current experiment, have generally not examined or reported changes in beta-band connectivity (Burke et al., 2013). However, changes in beta path length have been related to the progression of Alzheimer’s disease—namely, Alzheimer’s disease patients exhibit higher beta path lengths than healthy controls, and beta path length is negatively correlated to minimental state examination (MMSE) score (Stam et al., 2006). Additionally, research in the oscillatory neurophysiology of sleep has demonstrated that faster rhythmic activity (i.e., in the beta/gamma range) is present and sustained during the depolarization phase of the slow wave and largely absent during the hyperpolarization phase (Compte et al., 2008; Steriade, 2006; Steriade et al., 1996). This increased activity may be specifically linked to noradrenergic neurons in the locus coeruleus system (Eschenko et al., 2012), which may promote cortical plasticity, an important component of memory consolidation. Thus, it is a possibility that beta-band network organization and activity during SWOs may generally be an important aspect of episodic memory functioning and consolidation processes, which can be targeted and enhanced through specific STAMP stimulation.

One potential limitation of the current study is the lack of a control condition to rule out the overall effect of stimulation on connectivity. Namely, STAMPs could have been applied during sleep that were not tied to any episode during encoding to compare connectivity changes following tagged STAMPs to changes following untagged STAMPs. Given the limited number of STAMPs and complexity of the experimental design, we chose to maximize our statistical power by using all of the STAMPs to cue memory during sleep. However, this condition would have been particularly useful for understanding our classification results. Currently, we can say that the selected features can delineate the Active and Sham conditions, and this *may* be due to consolidation processes in the Active condition compared to the Sham condition; however, these may also be general changes following stimulation. In contrast, we are confident that the beta path length relationship to metamemory sensitivity changes is related to sleep-dependent consolidation processes. Our behavioral results show that STAMP stimulation led to benefits for the Tag & Cue condition compared to the other conditions, demonstrating a specific benefit as opposed to a more generic stimulation-induced benefit to metamemory. In line with this, changes in beta path length specifically predicted changes in Tag & Cue AUC changes, and was not predictive of sensitivity in the Sham condition.

Previous work examining brain connectivity with graph theoretic measures has often taken a more limited approach by examining specific measures or specific frequency bands of interest (Sadaghiani et al., 2015; Song et al., 2014). We chose to utilize a machine learning approach of searching through a large collection of measures in different frequency bands to identify significant changes following stimulation, and to use cross-validation and comparison to base models to validate our findings. However, this method is not without its limitations. The features selected may vary based on the algorithm or the set of processing steps chosen. Additionally, to greatly improve performance, we removed highly collinear features—however, these removed features could potentially still be of theoretical interest. We performed follow-up statistical tests to assure that our final set of selected features did statistically differ between the stimulation conditions and/or predict performance; nevertheless, there may be other connectivity features in certain bands that are also significantly modulated by STAMPs and/or are predictive of behavior, but simply were not selected by the algorithm. However, we believe that the beta path length relationship to behavior is robust, as it was consistently considered highly significant by the Boruta algorithm and significantly predicted the behavioral data, and this effect likely would not have been found had we only examined more standard measures and frequency bands. Future studies may identify the specific role beta network connectivity plays in episodic memory and decision-making processes.

## ACKNOWLEDGMENTS

This work was supported by the DARPA and the Army Research Office. The views, opinions and/or findings expressed are those of the author and should not be interpreted as representing the official views or policies of the Department of Defense or the United States Government.

## SUPPORTING INFORMATION

The connectivity features, as well as metamemory sensitivity values, are included in the Supporting Information for this article available at https://doi.org/10.1162/netn_a_00201.

## AUTHOR CONTRIBUTIONS

Ryan Hubbard: Conceptualization; Formal analysis; Methodology; Writing – original draft; Writing – review & editing. Iman Zadeh: Conceptualization; Formal analysis; Methodology; Resources; Software; Writing – original draft. Aaron Jones: Data curation; Resources. Bradley Robert: Data curation; Resources. Natalie Bryant: Data curation; Resources; Software. Vincent Clark: Data curation; Resources. Praveen Pilly: Conceptualization; Formal Analysis; Funding acquisition; Investigation; Project administration; Supervision; Writing – original draft; Writing - review & editing.

## FUNDING INFORMATION

Praveen Pilly, Defense Advanced Research Projects Agency (https://dx.doi.org/10.13039/100000185), Award ID: W911NF-16-C-0018.

## CONFLICT OF INTEREST STATEMENT

The last author, Praveen K. Pilly, has a patent on using transcranial electrical stimulation for targeted memory reactivation (US Patent No. 10,307,592). Vincent P. Clark is a scientific advisor with NeuroGeneces, LLC. All the authors declare that the research was conducted in the absence of any other competing financial or nonfinancial interests.

## TECHNICAL TERMS

Transcranial electrical stimulation (tES): Noninvasive brain stimulation technique in which electrical current flows from an anodal electrode to a cathode placed on the scalp. Small amounts of current pass through the skin and skull, reaching neurons in the brain and increasing membrane excitability.
Spatiotemporal amplitude-modulated pattern (STAMP): tES in which the distributed pattern of electrical stimulation across the scalp changes rapidly through time.
Electroencephalography (EEG): Brain imaging technique in which electrodes are placed on an individual’s scalp, allowing for recording of electrical activity propagated from the brain to the surface of the head.
Imaginary coherence: A measure of functional connectivity used in analysis of EEG data which uses the phase information of coherency. This method avoids the issue of volume conduction, which can highly inflate measures of connectivity, but also leads to a more conservative estimate of connectivity.
