# Supplementary material for: Brain connectivity alterations during sleep by closed-loop transcranial neurostimulation predict metamemory sensitivity
Source: Netw Neurosci. 2021 Aug 30;5(3):734–56. doi: 10.1162/netn_a_00201 (PMC8567828; doi:10.1162/netn_a_00201)
Supplement: Supplementary file 1 [file netn-05-734-s001.pdf]

Hubbard, R., Zadeh, I., Jones, A. P., Robert, B., Bryant, N. B., Clark, V. P. & Pilly, P. K. (2021). Supporting information for “ Brain connectivity alterations during sleep by closed-loop transcranial neurostimulation predict meta-memory sensitivity. *Network Neuroscience*. Advance publication. https://doi.org/10.1162/netn_a_00201

Supplementary Figure S1

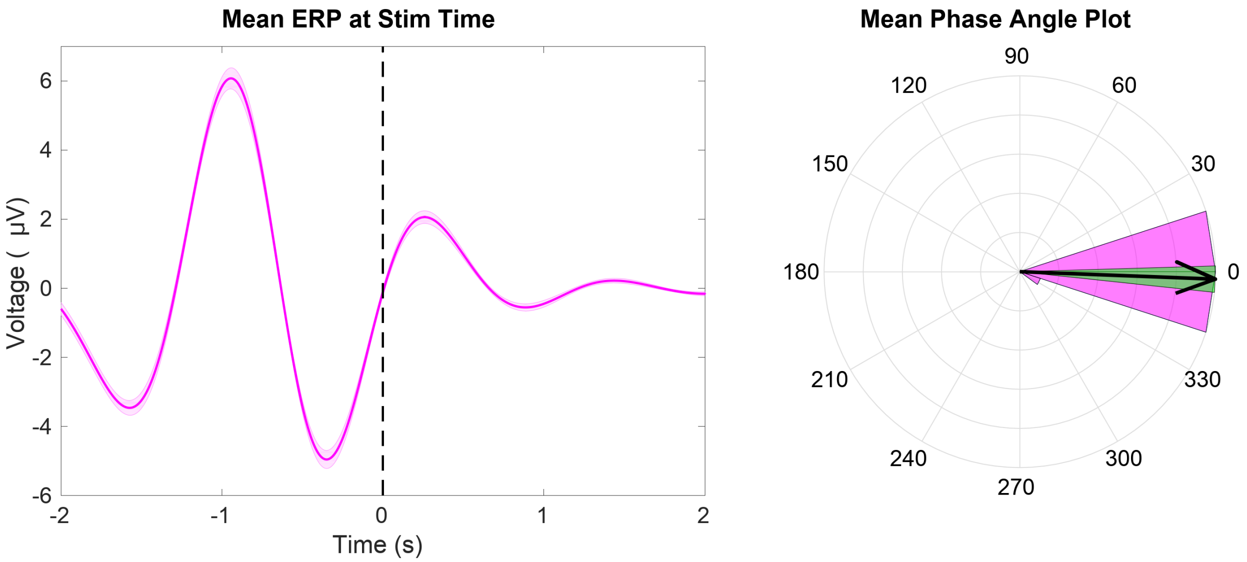

Closed-loop stimulation validation results. The mean ERP on the left plots the averaged voltage (µV) around stimulation timepoints across participants (shaded area shows 95% CI). Data is taken from the Sham condition. The dotted line marks the stimulation onset timepoint, right before the up-state. The phase angle plot on the right shows the distribution of average phase values at the stimulation onset timepoint across participants (the pink shaded region), with the arrow marking the grand average. Green shaded region shows the 95% CI.

Supplementary Figure S2

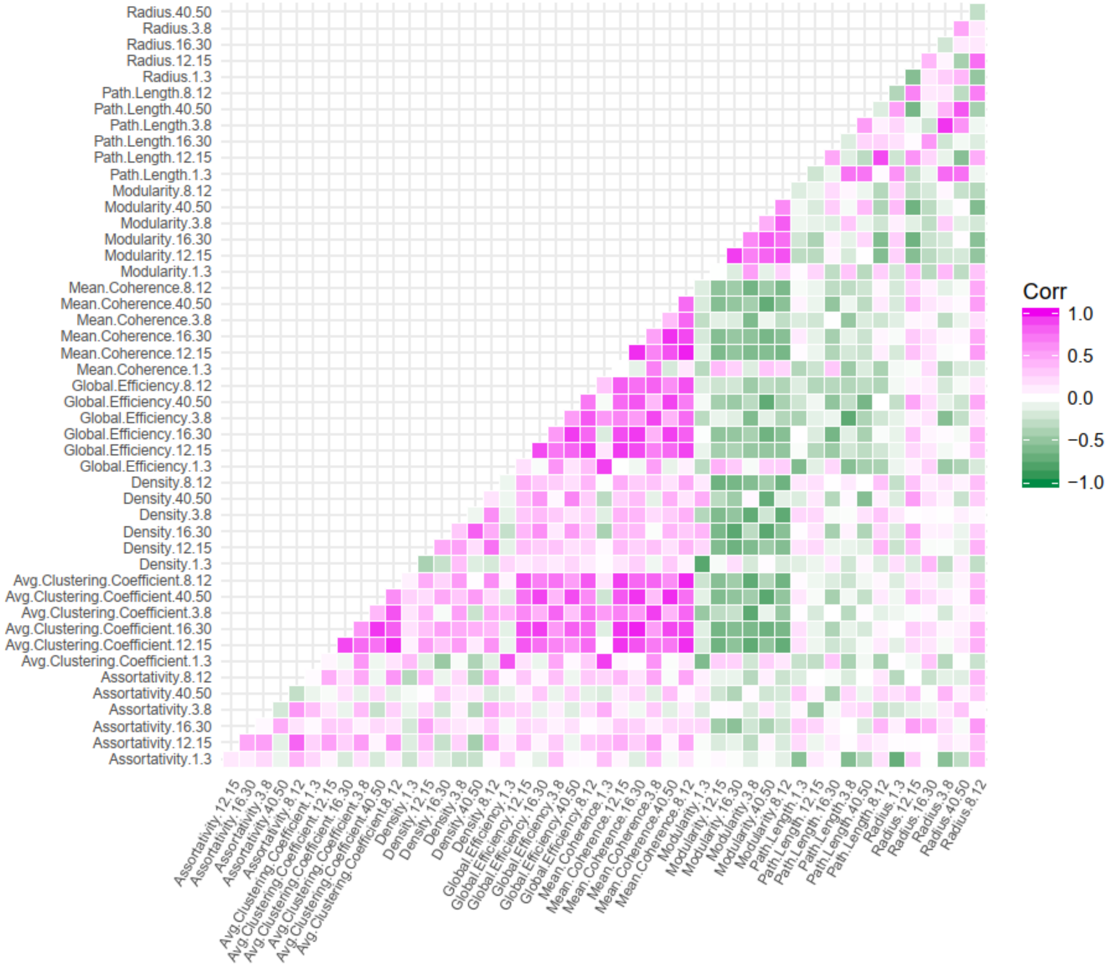

Correlations between graph theoretic features in different frequency bands. The figure shows correlations between measures across participants in the Active condition. Magenta colors represent positive correlations, green colors represent negative correlations. High collinearity is observed, particularly between similar features in different frequencies (e.g. Mean Coherence).

Supplementary Figure S3

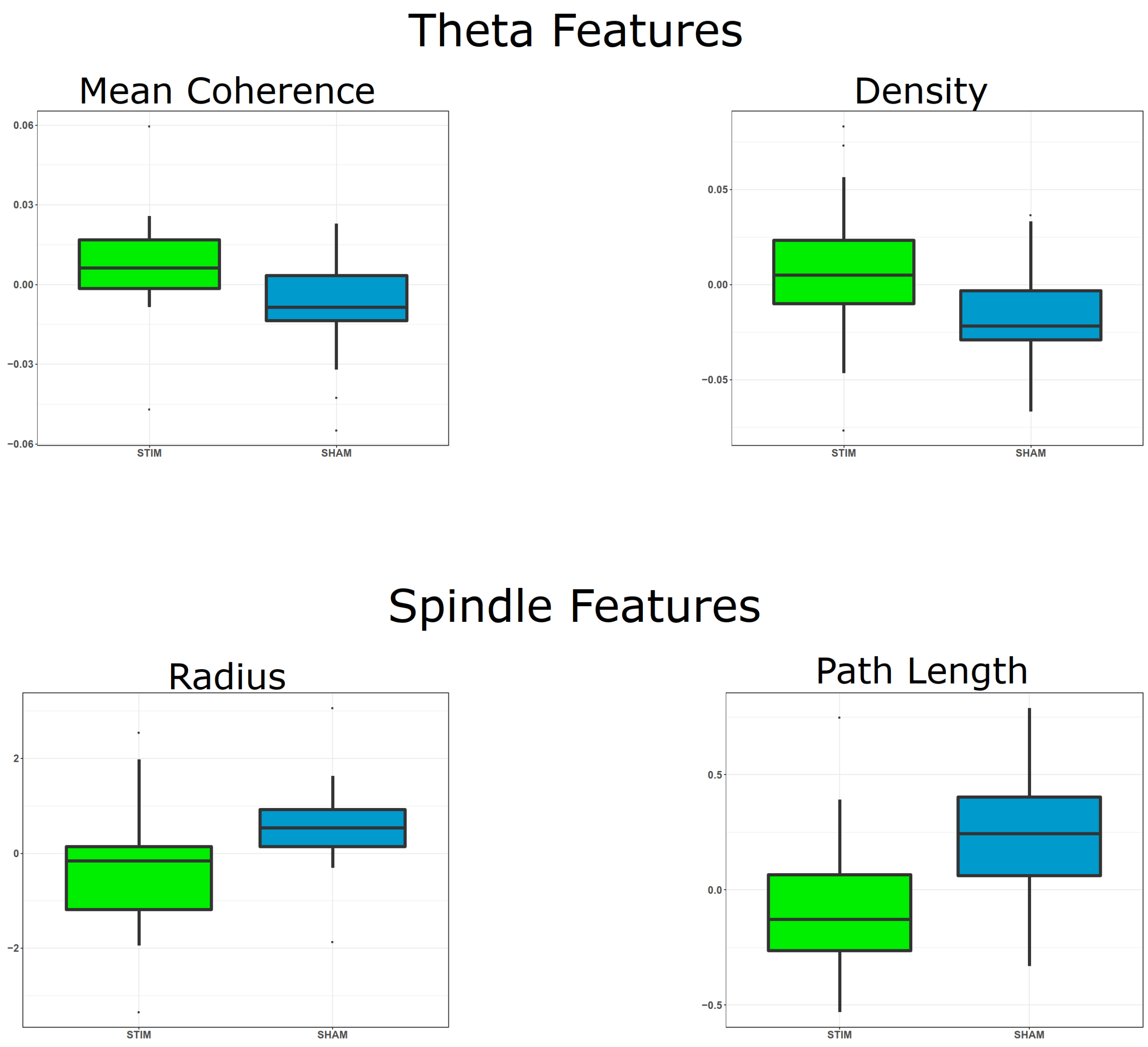

Graph theoretic features selected by classification analysis. Follow-up t-tests, corrected with a false discovery rate, verified that each feature significantly differed between Active (green) and Sham (blue) conditions. Theta (3-8 Hz) Density, $p = 0.05$; all other features (Theta Mean Coherence, Spindle (12-15 Hz) Radius and Path Length), $p = 0.029$. Active stimulation was associated with increases in Theta Coherence and Density measures, as well as decreases in Spindle connectivity measures, suggesting increased efficiency of communication in the network.

Supplementary Figure S4

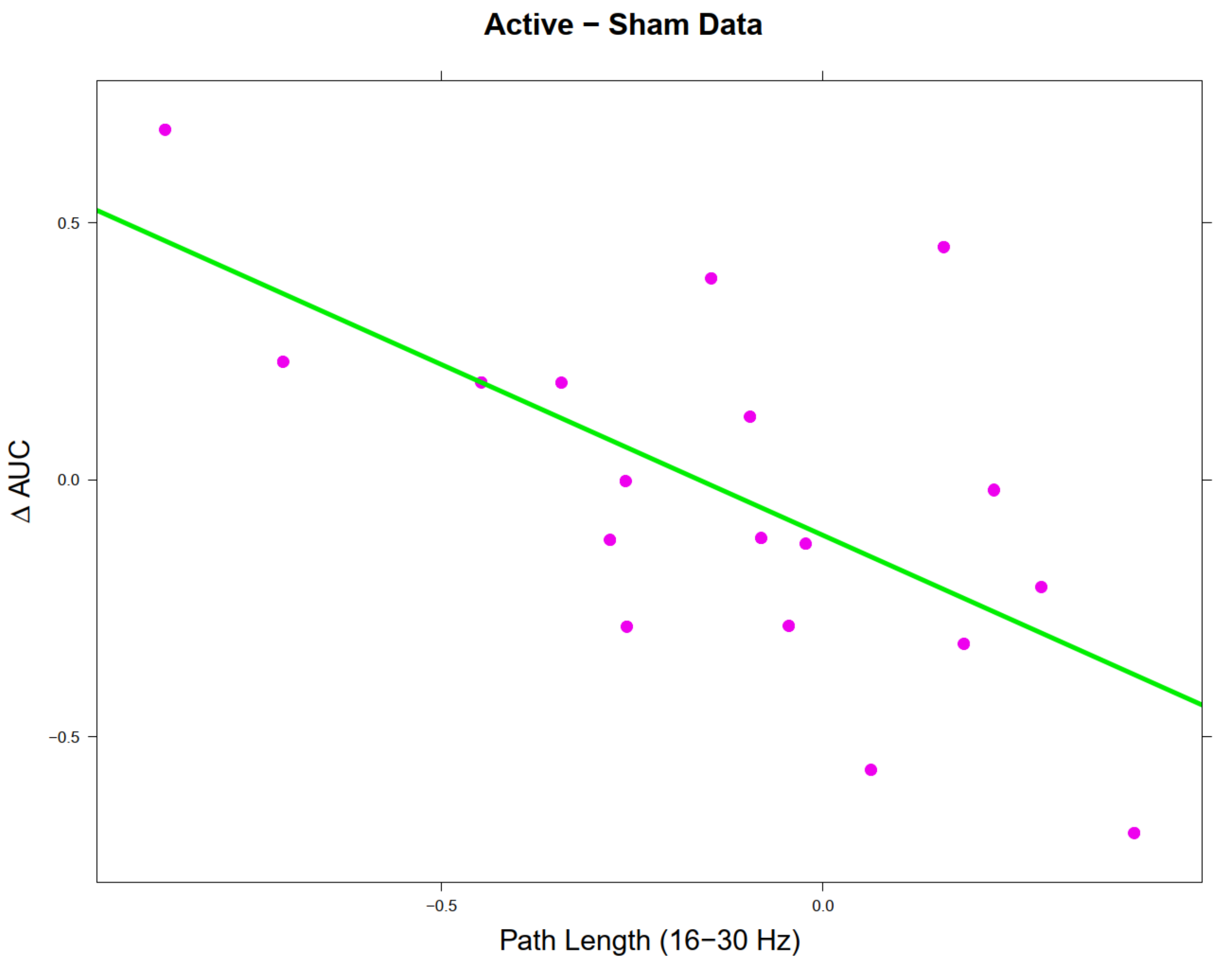

Sham subtraction analysis. The plot shows the regression of Beta Path Length on change in pre to post-sleep AUC for the data in which the Sham data was subtracted from the Active data for both the Beta Path Length and AUC variables. Beta Path Length is still a significant predictor in the analysis (t = -3.27, p = 0.005, adj. R^2 = 0.36).

Supplementary Figure S5

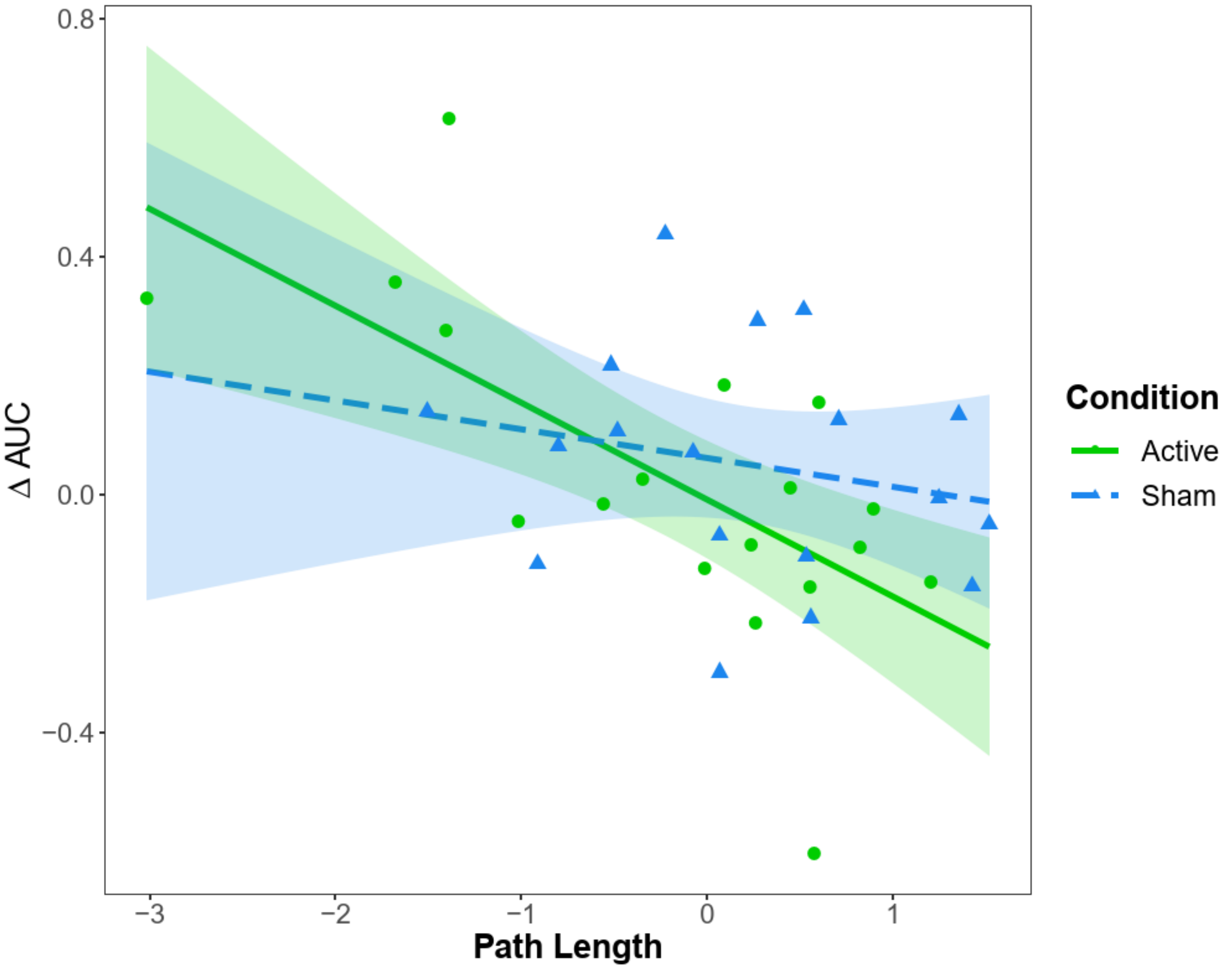

Interaction between Beta Path Length and stimulation condition. The relationship between Beta Path Length and AUC change is significant in the Active condition (t = -3.56, p = 0.003, adj. R^2 = 0.41), not significant for the Sham condition (t = -0.88, p = 0.39, adj. R^2 = -0.01). However, the interaction between Beta Path Length and stimulation condition only trended toward significance (t = 1.69, p = 0.1).

Supplementary Figure S6

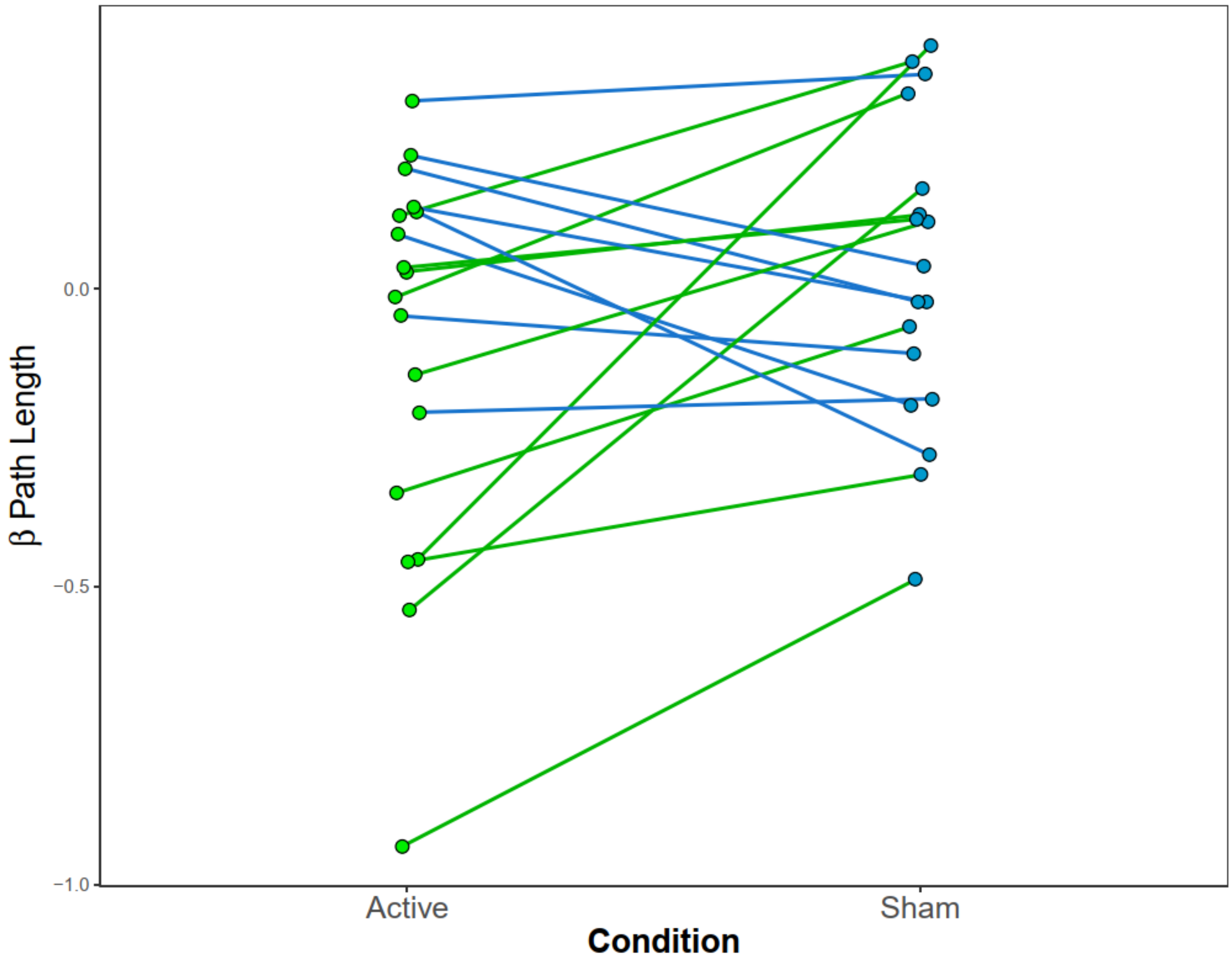

Individual differences in Path Length modulation. Each subject's Beta Path Length in both the Active and Sham conditions is plotted above, with a connecting line to demonstrate the slope of change. Individuals that showed a beneficial response (a decrease from Sham to Active) are plotted in green, while those that show a detrimental response (an increase from Sham to Active) are plotted in blue.

Supplementary Note 1. Closed-Loop Stimulation Validation

The grand average ERP centered around the stimulation onset timepoint, as well as the mean phase angles at the stimulation onset timepoint across participants, are plotted in Figure S1. There was high consistency across participants; the mean of the participant mean phase angles was -2.18 degrees (± 6 degrees), which did not significantly differ from 0 degrees (v-test v = 18.8, p < 0.001). We successfully targeted up-states of slow-wave oscillations with our closed-loop system.

Supplementary Note 2. Additional Information on Correlated Variables

As part of the pipeline of the implemented machine learning analyses, highly collinear variables were removed by finding pairs of features with high correlations (threshold r value of 0.8) and removing the variable with the higher mean absolute correlation with the other variables. Across folds, an average of 22 features were removed due to high collinearity. The removed features were largely those with high within-category correlations (see Supplementary Figure 2). These included Average Clustering Coefficient, Mean Coherence, and Global Efficiency. These features were likely often removed due to their high correlations across categories; for example, in the Alpha band, Mean Coherence and Average Clustering Coefficient were highly correlated (r = 0.96), as well as Mean Coherence and Global Efficiency (r = 0.85). Path Length in the Theta and Alpha bands were also often removed. Radius, Density, and Assortativity features were infrequently removed.

Post-hoc exploratory statistical tests were performed to compare the features selected as most important from the machine learning analysis to the removed colinear features. Mean Coherence in the Theta band was significantly correlated with Mean Coherence features in other frequency bands (Alpha, r = 0.77; Spindle, r = 0.65; Beta, r = 0.50). Similarly, significant correlations were found between Theta band Mean Coherence and Average Clustering Coefficient, as well as Global Efficiency features in other frequency bands. This result suggests that these features were likely influence by a common underlying source of variance, and that stimulation influenced this connectivity broadly across frequency bands. This was not the case for Theta band Density, which was not significantly correlated with Mean Coherence, Average Clustering Coefficient, or Global Efficiency features in other frequencies.

For Path Length features, a strong correlation was found between Alpha and Spindle band Path Length (r = 0.88), while weaker correlations were found between other frequency bands (Spindle and Beta, r = 0.48; Spindle and Theta, r = -0.15). To confirm the selectivity of Beta band Path Length in

predicting change in meta-meory sensitivity, we ran linear regression models predicting change in AUC with Path Length features in other frequency bands. No other Path Length feature significantly predicted behavioral performance change (Spindle, $p = 0.15$; Alpha, $p = 0.29$; Theta, $p = 0.80$). Thus, while Alpha and Spindle Path Length were highly correlated, potentially reflecting contributions of slow and fast spindles, Beta Path Length exclusively predicted behavioral performance changes.

Supplementary Note 3. Additional Sham Analyses

We performed additional analyses with the Sham data in order to provide further support for the notion that the effects reported in this study were as a result of transcranial current stimulation. Firstly, as reported in the Results section, we looked for relationships between graph theoretic connectivity measures identified in the Active data (namely, Beta Path Length) and pre to post sleep AUC in the Sham data with a linear regression. This was not significant (r = -0.22, p = 0.39). As an additional step, we entered the Sham data connectivity measures into the Boruta algorithm to see if Beta Path Length, or potentially other measures, would be identified. Consistently, the Boruta algorithm returned no features that were more informative than the added noise features. For a follow-up analysis, we performed a multivariate permutation test of correlations between the graph theoretic measures from the Sham data and overnight AUC change, in which correlations between each measure and AUC change were computed and statistically compared to a distribution obtained by repeatedly shuffling and computing correlation statistics [1]. No significant correlations were found. Thus, it appeared that the relationship between Beta Path Length and memory consolidation was only observed in the Active condition, not in the Sham condition.

An alternative analysis method would be to subtract the Sham data from the Active data as a sort of baseline correction, here called Active-Sham data. We performed the same linear regression predicting change in AUC performance with Beta Path Length, but with the Active-Sham data. The regression is illustrated in Supplementary Figure S4, and the relationship remains significant (t = -3.27, p = 0.005, adj. R^2 = 0.36).

Given the significant relationship in the Active condition and the non-significant relationship in the Sham condition, one may expect a significant interaction between Beta Path Length and stimulation condition in predicting AUC change. The interaction is illustrated in Supplementary Figure S5. However,

a linear model with an interaction term reported no significant interaction (t = 1.69, $p = 0.1$). This non-significant interaction could mean that stimulation was not related to the Beta Path Length relationship with behavior that was observed, and in fact this was potentially identified spuriously.

We would argue that the classification analysis identified multiple connectivity measures that significantly differed between the Active and Sham conditions, suggesting stimulation did have a measurable impact on neural connectivity. Second, the previously discussed analyses demonstrate that the variability in connectivity measures in the Sham data was not predictive of behavior change, and in fact no features were selected by the Boruta algorithm; however, Beta Path Length was consistently selected as significant in the Active data, and was generally considered much more informative than the noise features. Finally, in comparing changes in Beta Path Length between the Active and Sham conditions in each subject, it is clear that there are individual differences in response to stimulation, as illustrated in Supplementary Figure S6. Some participants had very little change in Path Length or even an increase in Path Length in the Active condition compared to the Sham condition. However, these changes were not large in magnitude, and thus detecting this potentially subtle cross-over interaction may simply require more statistical power.

Supplementary References

[1] Yoder, P. J., Blackford, J. U., Waller, N. G., & Kim, G. (2004). Enhancing power while controlling family-wise error: an illustration of the issues using electrocortical studies. *J Clin Exp Neuropsych*, 26(3), 320-331.
